# Value of Thromboelastography as a Predictor of Postoperative Acute Respiratory Distress Syndrome in Patients With Acute Type A Aortic Dissection

**DOI:** 10.31083/RCM47295

**Published:** 2026-04-21

**Authors:** Xiujuan Wang, Kailong Ye, Xingfeng Chen, Yurou Guo, Beiran You, Zhihuang Qiu, Qingsong Wu

**Affiliations:** ^1^Department of Cardiovascular Surgery, Fujian Medical University Union Hospital, 350001 Fuzhou, Fujian, China; ^2^Department of Respiratory and Critical Care Medicine, Mindong Hospital Affiliated to Fujian Medical University, 352101 Ningde, Fujian, China; ^3^Fujian Medical University, 350005 Fuzhou, Fujian, China

**Keywords:** aortic dissection, respiratory distress syndrome, thromboelastography, blood coagulation, predictive value of tests

## Abstract

**Background:**

This study aimed to evaluate the effectiveness of thromboelastography (TEG) in predicting postoperative acute respiratory distress syndrome (ARDS) in patients with acute type A aortic dissection (ATAAD).

**Methods:**

This retrospective cohort study included 350 consecutive patients with ATAAD who underwent emergency total aortic arch replacement surgery at our institution. Patients were divided into ARDS and non-ARDS groups based on the postoperative development of ARDS. Perioperative data were collected and compared between groups.

**Results:**

Overall, 56/350 (16.0%) patients developed postoperative ARDS, of whom four required reintubation and four progressed to respiratory failure. Although 30-day mortality was similar between the ARDS and non-ARDS groups (8.9% vs. 4.1%; *p* = 0.227), postoperative complications were more complex and severe in the ARDS group. Indeed, this group had longer ventilator use (*p* = 0.009), a higher incidence of severe pneumonia (*p* = 0.026), longer intensive care stays (*p *= 0.019), and more frequent respiratory failure (*p* = 0.096) and multiple organ failure (*p* = 0.040). Multivariate analysis identified preoperative clot angle (odds ratio (OR) 4.421, 95% confidence interval (CI) 1.922–8.743; *p* < 0.001), fibrinogen level (OR 4.473, 95% CI 2.678–9.399; *p* < 0.001), maximum amplitude (MA) (OR 4.552, 95% CI 2.089–8.947; *p* < 0.001), cardiopulmonary bypass time (OR 2.796, 95% CI 1.166–6.705; *p* = 0.021), and intraoperative plasma transfusion (OR 4.057, 95% CI 1.700–9.046; *p* = 0.004) as independent predictors of postoperative ARDS. The optimal cut-off values for preoperative fibrinogen level, clot angle, and platelet function (MA) on the receiver operating characteristic (ROC) curve analysis were 2.65 μg/mL, 59.4 degrees, and 64.1 mm, respectively, with corresponding areas under the curve of 0.744, 0.781, and 0.807 (all *p* < 0.001).

**Conclusion:**

Preoperative fibrinogen, clot angle, and MA may be useful predictors of postoperative ARDS in patients with ATAAD. TEG enables rapid preoperative assessment of coagulation system status, guiding fibrinogen supplementation and blood transfusion strategies to reduce the incidence of postoperative ARDS and shorten the duration of postoperative mechanical ventilation. Thus, TEG may be a valuable tool for real-time monitoring and improving postoperative outcomes in this population.

## 1. Introduction

Acute type A aortic dissection (ATAAD) is an extremely high-risk aortic disease, with a very high early mortality rate, for which the only treatment strategy is surgery, preferably performed as early as possible. Indeed, early surgical repair has been associated with improved prognosis and reduced mortality [1]. Acute respiratory distress syndrome (ARDS) is a common and serious complication in patients with ATAAD following total arch replacement. This complication is associated with high rates of morbidity and mortality [2], which seriously affect the postoperative prognosis and survival rate of these patients [3, 4, 5]. Indeed, in contrast to other cardiovascular operations, ATAAD surgery requires low-temperature and low-flow perfusion, or even circulatory arrest, which can lead to an imbalance in the body’s inflammatory and coagulation systems [6]. Activation of the clotting system is currently considered one of the key factors influencing progression and prognosis in patients with ATAAD [7], while excessive perioperative bleeding and transfusion have been identified as independent risk factors of ARDS after ATAAD [8]. Aortic dissection triggers activation of the coagulation/fibrinolytic system and platelets and the high consumption of coagulation factors and platelets, resulting in increased intraoperative bleeding, one of the most common complications of ATAAD [9, 10]. Consequently, the incidence of postoperative ARDS is among patients with ATAAD.

Early detection and the timely prevention of potential risk factors of postoperative ARDS are important to improve the overall prognosis of patients with ATAAD. However, traditional laboratory tests often fail to accurately reflect and evaluate the overall preoperative coagulation function among patients with ATAAD. As such, rapid identification of the preoperative coagulation function status and coagulation reserve status of patients with TAAD is imperative. Based on these measurements, individualized fibrinogen, coagulation factor, and platelet supplements can be administered to optimize blood transfusion and reduce the occurrence of postoperative ARDS, to improve the overall therapeutic effect and prognosis of patients with TAAD.

## 2. Materials and Methods

### 2.1 Patient Population

Patients diagnosed with ATAAD who underwent total aortic arch replacement surgery between January 2020 and December 2022 at the Fujian Medical University Union Hospital were retrospectively included and divided into ARDS and non-ARDS groups, according to whether they developed ARDS after surgery. We used the results of preoperative (within 48 h) routine laboratory tests and thromboelastography (TEG) analysis to analyze the relationship between the coagulation system and postoperative ARDS in the 350 enrolled patients with ATAAD who underwent the emergency total aortic arch replacement procedure. This study was approved by the Ethics Committee of our Institute. Informed consent was waived in accordance with institutional policy for retrospective studies.

**Fig. 1.**
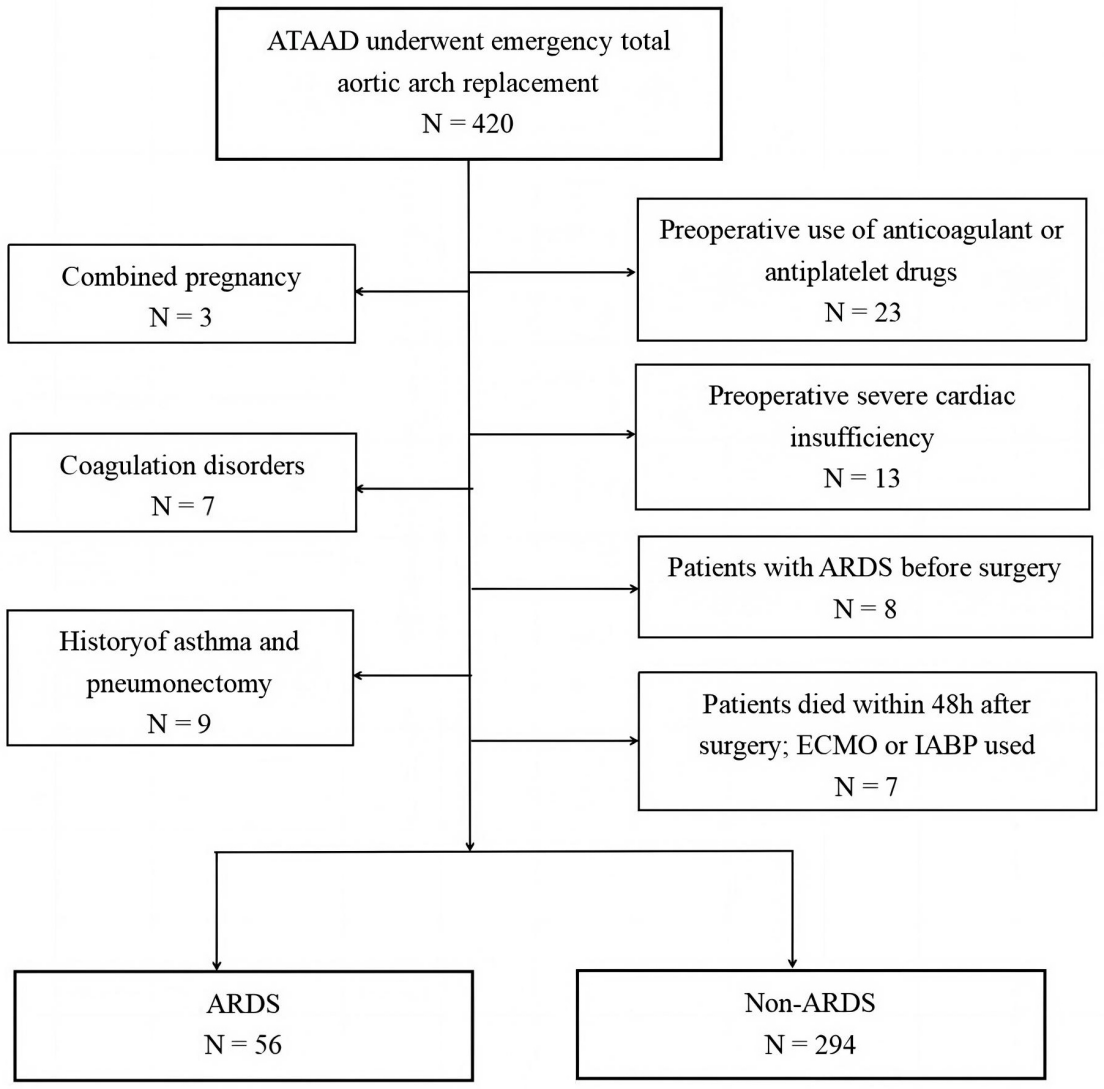
**Flowchart of the study cohort**. ARDS, acute respiratory distress syndrome; ATAAD, acute type A aortic dissection; ECMO, extracorporeal membrane oxygenation; IABP, intra-aortic balloon pump.

The inclusion criteria were as follows: all patients with ATAAD who underwent total aortic arch replacement within 48 h of onset. The exclusion criteria were as follows: (1) patients with a congenital or acquired coagulation disorder, abnormal liver function, or severe liver disease; (2) anticoagulant or antiplatelet drugs used before surgery; (3) preoperative severe cardiac insufficiency (New York Heart Association (NYHA) grade ≥III, left ventricular ejection fraction (LVEF) <40%); (4) patients with other serious lung diseases associated with ARDS prior to surgery, including chronic obstructive pulmonary disease, interstitial pneumonia, silicosis, pneumoconiosis, or lung cancer, among others; (5) a history of asthma and pneumonectomy; (6) death or a requirement for postoperative mechanical support, including an intra-aortic balloon pump or extracorporeal membrane oxygenation within 48 h of surgery (Fig. 1). For individual instances of lost data, we used the mode principle of statistics to replace the missing values. For instances where there were several missing values (more than three), we omitted the case.

### 2.2 Measurements and Clinical Data

Comprehensive serum examinations, including complete blood counts, a full biochemistry panel, fibrinogen levels, blood gas analysis, and TEG analysis, were performed for all patients after admission. The clinical data collected included the baseline, intraoperative, and postoperative outcomes. In patients with persistent hypoxemia, chest radiography, chest computed tomography, and bronchoalveolar lavage bronchoscopy were performed to rule out other causes of postoperative hypoxemia. Cardiac function was evaluated using bedside echocardiography to rule out pulmonary dysfunction caused by perioperative heart failure factors to ensure the accuracy of ARDS.

### 2.3 Relevant Indicators of TEG

The relevant indicators of TEG were as follows: (1) reaction time (R-time), this parameter indicates the delay in the onset of fibrin formation, with a prolonged R-time signifying a slower initiation of clotting; (2) kinetic time (K-time), the K-time quantifies the rate at which the clot forms, where an extended K-time suggests a delayed progression in clot maturation; (3) clot angle, this value reflects the speed of fibrin crosslinking during clot formation, where a smaller clot angle denotes a slower rate of fibrin network development; (4) maximum clot firmness, this measure provides insight into the overall strength of the clot, where a lower maximum clot firmness indicates a clot with diminished stability and strength.

### 2.4 Definitions

Hypoxemia is a condition in which the level of oxygen in the blood is insufficient, resulting in a lower-than-normal arterial partial pressure of oxygen (PaO_2_) compared to normal levels in individuals of the same age. The normal range of the arterial PaO_2_ is 90–100 mmHg, whereas hypoxemia is diagnosed when the pressure drops below 60 mmHg. The severity of hypoxemia is generally measured based on the oxygenation index (OI, PaO_2_/FiO_2_), whereas OI values ≤300 mmHg indicate hypoxemia, excluding patients with transient hypoxemia [3, 11]. There are two types of hypoxemia, specifically isolated hypoxemia and ARDS [3].

ARDS was defined according to Berlin’s definition [12]. In brief, it was diagnosed in patients with an OI ≤300 mmHg within 7 days after surgery; however, the extent of lung injury required an assessment based on chest radiography or chest computed tomography. If necessary, bronchoalveolar lavage bronchoscopy or a pulmonary artery catheter was performed to evaluate the pulmonary artery wedge pressure (PAWP) to rule out any other causes of postoperative hypoxemia. Further, cardiac function was evaluated via bedside echocardiography to rule out cardiogenic pulmonary edema. Patients with ARDS were classified into three categories according to severity: mild (200 mmHg < OI ≤ 300 mmHg), moderate (100 mmHg < OI ≤ 200 mmHg), and severe (OI ≤100 mmHg) [12].

### 2.5 Statistical Analysis 

SPSS 24.0 (IBM, Armonk, NY, USA) was used for statistical analysis. Categorical variables are presented as the n value (%), and Fisher exact or Chi-square tests were used for comparisons. According to whether the continuous variables conformed with normality, they were expressed based on the means ± standard deviations, or the quartile method and *T*-tests or U-tests were performed for comparisons. The absence of multicollinearity among the key coagulation variables was confirmed through a diagnostic evaluation, suggesting that these variables were not collinear with one another. Receiver operating characteristic (ROC) curves were used to evaluate the predictive power of the serious risk factors for postoperative ARDS. Continuous variables were also dichotomized based on optimal cut-off values determined based on the ROC curve analysis for use in logistic regression models. Univariate analysis was applied to compare the baseline characteristics of the two groups, and a multifactor stepwise logistic regression model was used to identify the possible risk factors for postoperative ARDS (*p* < 0.05). For all analyses, a two-tailed value of *p* < 0.05 was considered statistically significant.

## 3. Results

### 3.1 Baseline Characteristics

The patients were divided into two groups (ARDS and non-ARDS) according to whether ARDS occurred after surgery. The baseline demographic information of the patients and results of routine preoperative laboratory tests are shown in Table 1. The kinetic times in the ARDS group were higher than those in the non-ARDS group (2.1 ± 0.9 vs. 1.5 ± 0.6, *p* < 0.001). However, the clot angle (59.6 ± 10.4 vs. 66.2 ± 8.0, *p* < 0.001), maximum amplitude (MA) (58.2 ± 9.2 vs. 65.4 ± 8.9, *p* < 0.001), fibrinogen level (3.1 ± 1.3 vs. 3.6 ± 1.5, *p* = 0.013), and platelet level (178.1 ± 45.0 vs. 199.4 ± 62.1, *p *= 0.003) were lower in the ARDS group than in the non-ARDS group.

**Table 1.** **Comparison of baseline data between the two patient groups**.

Variables		ARDS group (n = 56)	Non-ARDS group (n = 294)	*p*-value
Male, n (%)		35 (62.5)	192 (65.3)	0.687
Age, n (years)		51.5 (45.0, 61.5)	52.0 (43.0, 60.0)	0.444
Body mass index (kg/m^2^)		25.7 ± 3.8	25.7 ± 4.0	0.972
Hypertension, n (%)		43 (76.8)	221 (75.2)	0.797
Diabetes, n (%)		6 (10.7)	27(9.2)	0.719
Coronary heart disease, n (%)		5 (8.9)	30 (10.2)	0.771
Marfan syndrome		2 (3.6)	9 (3.1)	1.000
History of cardiac surgery, n (%)		4 (7.1)	15 (5.1)	0.767
Malperfusion syndrome, n (%)				
	Penn A (no malperfusion)	50 (89.3)	270 (91.8)	0.715
	Penn Ab/c/bc (any malperfusion)	6 (10.7)	24 (8.2)	0.715
Thromboelastography				
	Reaction time (min)	6.2 ± 1.2	5.9 ± 1.1	0.098
	Kinetics time (min)	2.1 ± 0.9	1.5 ± 0.6	<0.001
	Clot angle (deg)	59.6 ± 10.4	66.2 ± 8.0	<0.001
	MA (mm)	58.2 ± 9.2	65.4 ± 8.9	<0.001
Fibrinogen (µg/mL)		3.1 ± 1.3	3.6 ± 1.5	0.013
Prothrombin time (s)		14.3 ± 1.8	14.3 ± 2.4	0.885
International normalized ratio		1.17 ± 0.41	1.22 ± 1.15	0.743
D-Dimer (µg/mL)		10.2 (6.5, 18.4)	7.9 (3.5, 18.4)	0.072
Leukocyte (10^9^/L)		12.0 ± 4.0	11.0 ± 3.2	0.046
Platelet (10^9^/L)		178.1 ± 45.0	199.4 ± 62.1	0.003
Hemoglobin (g/L)		131.5 ± 21.4	129.1 ± 21.0	0.432
Lactic acid (mmol/L)		1.9 ± 1.2	1.8 ± 1.3	0.572
Alanine aminotransferase (IU/L)		29.0 (21.0, 53.5)	26.0 (20.0, 43.0)	0.451
Aspartate aminotransferase (IU/L)		27.5 (19.5, 67.0)	27.0 (19.0, 44.0)	0.467
Albumin (g/L)		36.8 ± 4.7	37.7 ± 4.9	0.223
Serum creatinine (µmol/L)		81.3 (66.3, 117.0)	82.5 (65.0, 124.0)	0.621
LVEF (%)		63.4 ± 6.7	63.7 ± 7.0	0.775

Continuous normally distributed variables were expressed as the mean ± standard deviation ($\bar{x}$ ± s), with non-normally distributed variables as medians (interquartile range). Counts are expressed as percentages. The χ^2^ test was used for categorical variables, and the Wilcoxon rank sum test was used for continuous variables. MA, maximum amplitude; LVEF, left ventricular ejection fraction.

### 3.2 Incidence of Postoperative ARDS in Patients With ATAAD

Based on the ARDS evaluation criteria, the incidence of ARDS after ATAAD was 16.0% (56/350). The mean age of patients in the ARDS group was 51.5 years, and there were 35 men and 21 women. The 30-day mortality rate in this group was 8.9% (five cases), whereas a further four patients each (7.1%) required reintubation and developed postoperative respiratory failure.

### 3.3 Operative and Postoperative Data

The intraoperative and postoperative patient data are presented in Table 2. Overall, cardiopulmonary bypass (CPB) and aortic cross-clamp times were prolonged in patients with ARDS (*p* < 0.001 and *p* = 0.001, respectively), whereas intraoperative red blood cell infusion and intraoperative plasma transfusion were increased in the ARDS group (*p* = 0.008 and *p* < 0.001). Although the 30-day mortality rates were similar between the groups (8.9% vs. 4.1%, *p* = 0.227), postoperative complications were more complex and severe in the ARDS group. Indeed, this group was associated with prolonged ventilator use [44.5 (29.5, 69.0) vs. 36 (24.0, 58.0), *p* = 0.009], a higher incidence of severe pneumonia (12.5% vs. 4.1%, *p *= 0.026), longer intensive care times [5.0 (3.0, 8.5) vs. 4.0 (3.0, 6.0), *p* = 0.019], a higher rate of respiratory failure (7.1% vs. 2.0%, *p* = 0.096), and a higher rate of multiple organ failure (10.7% vs. 3.4%, *p* = 0.040).

**Table 2.** **Surgical and postoperative data on the two patient groups**.

Variables		ARDS group (n = 56)	Non-ARDS group (n = 294)	*p*-value
Operative time (min)		303.7 ± 52.8	299.9 ± 55.4	0.599
CPB time (min)		153.2 ± 39.7	135.9 ± 32.0	<0.001
Aorta cross-clamp time (min)		57.3 ± 20.1	48.1 ± 17.7	0.001
SCP time (min)		13.4 ± 4.7	12.8 ± 4.2	0.311
Concomitant procedure				
	Bentall, n (%)	12 (21.4)	88 (29.9)	0.197
	Aortic valve repair, n (%)	10 (17.9)	50 (17.0)	0.877
	CABG, n (%)	7 (12.5)	43 (14.6)	0.677
Intraoperative blood loss (mL)		376.3 ± 127.4	369.9 ± 133.9	0.687
Intraoperative red blood cells transfusion (U)		3.0 ± 1.7	2.3 ± 1.6	0.008
Intraoperative plasma transfusion (mL)		226.8 ± 179.4	108.5 ± 130.5	<0.001
30-day mortality, n (%)		5 (8.9)	12 (4.1)	0.227
Re-thoracotomy for hemostasis, n (%)		2 (3.6)	4 (1.4)	0.247
Pericardial drainage volume in 24 h after surgery (mL)		376.8 ± 208.8	398.2 ± 231.6	0.519
Mechanical ventilation time (h)		44.5 (29.5, 69.0)	36 (24.0, 58.0)	0.009
Re-intubation, n (%)		4 (7.1)	3 (1.0)	0.013
Intensive care time (d)		5.0 (3.0, 8.5)	4.0 (3.0, 6.0)	0.019
Hospitalization time (d)		18.5 (13.0, 24.5)	18.0 (13.0, 23.0)	0.856
Acute kidney failure, n (%)		5 (8.9)	19 (6.5)	0.703
Severe pneumonia, n (%)		7 (12.5)	12 (4.1)	0.026
Sepsis, n (%)		3 (5.4)	5(1.7)	0.234
Respiratory failure, n (%)		4 (7.1)	6 (2.0)	0.096
Multiple organ failure, n (%)		6 (10.7)	10 (3.4)	0.040

Continuous normally distributed variables are expressed as the mean ± standard deviation ($\bar{x}$ ± s), and not-normally distributed variables are expressed as medians (interquartile range). Counts are expressed as percentages. The χ^2^ test was used for categorical variables, and the Wilcoxon rank sum test was used for continuous variables. CPB, cardiopulmonary bypass; SCP, selective cerebral perfusion; CABG, coronary artery bypass grafting.

### 3.4 Changes in the Preoperative Fibrinogen Level, Clot Angle, and MA With Severity in the ARDS Group

**Fig. 2.**
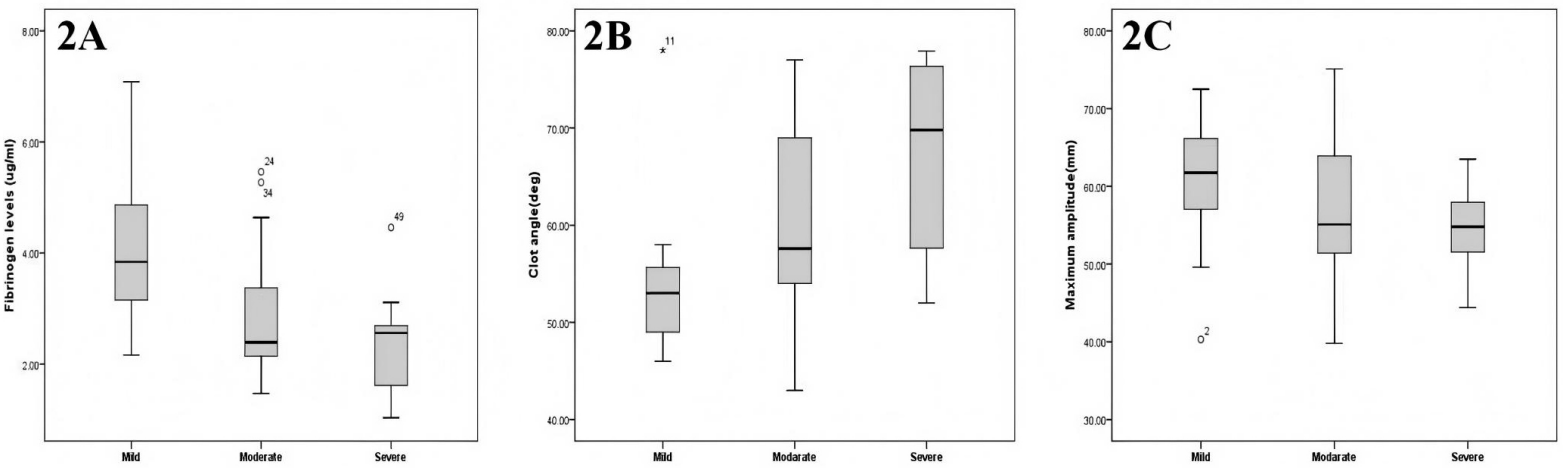
**Changes in the preoperative fibrinogen, clot angle, and MA levels corresponding to the different severities of postoperative ARDS**. (A) Preoperative fibrinogen levels of patients with mild, moderate and severe ARDS. (B) Preoperative clot angle of patients with mild, moderate and severe ARDS. (C) Preoperative maximum MA level of Patients with mild, moderate and severe ARDS.

In the overall trend analysis, we observed a significant difference in preoperative fibrinogen levels between patients with mild and moderate ARDS based on a classification comprising the OI decline (*p* = 0.016) (Fig. 2A). Similar to those in the overall analysis, we identified significant differences in the preoperative clot angle (fibrinogen function) across the mild, moderate, and severe ARDS groups in the overall trend analysis (*p* = 0.001) (Fig. 2B). In addition, in the overall trend analysis of patients with ARDS, the preoperative maximum MA level was increasingly lower in the mild, moderate, and severe ARDS groups (*p* = 0.036) (Fig. 2C).

### 3.5 Univariate and Multivariate Logistic Regression Analyses of Independent Risk Factors for Postoperative ARDS

In the univariate analysis, preoperative indices such as the D-dimer level, platelet count, fibrinogen level, kinetics time, clot angle, and MA level were all correlated with postoperative ARDS, indicating that they are risk factors (*p* < 0.05). Univariate analysis also showed that the CPB time, aortic cross-clamp time, selective cerebral perfusion time, intraoperative plasma transfusion, and red blood cell transfusion were all associated with the risk of postoperative ARDS (*p* < 0.05). To solve the collinearity problem, we conducted multivariate analysis to identify risk factors for ARDS. Overall, the univariate analysis identified preoperative fibrinogen level (odds ratio [OR], 4.473; 95% confidence interval [CI], 2.678–9.399; *p* < 0.001), clot angle level (OR, 4.421; 95% CI, 1.922–8.743; *p* < 0.001), MA level (OR, 4.552; 95% CI, 2.089–8.947; *p* < 0.001), CPB time (OR, 2.796; 95% CI, 1.166–6.705; *p* = 0.021), and intraoperative plasma transfusion (OR, 4.057; 95% CI, 1.700–9.046; *p* = 0.004) as independent risk factor for postoperative ARDS in patients with ATAAD (Table 3).

**Table 3.** **Results of univariate and multivariate logistic regression analyses of risk factors for postoperative ARDS in patients with ATAAD**.

Variable	Univariate model			Multivariate model		
	OR	95% CI	*p*-value	OR	95% CI	*p*-value
Age ≥55, years	0.908	0.510–1.617	0.743	-	-	-
Male sex	0.885	0.490–1.600	0.687	-	-	-
Body mass index ≥25.0 (kg/m^2^)	1.388	0.767–2.512	0.279	-	-	-
Hypertension	1.093	0.557–2.145	0.797	-	-	-
Diabetes mellitus	1.187	0.466–3.022	0.720	-	-	-
Coronary artery disease	0.863	0.320–2.329	0.771	-	-	-
History of cardiac surgery	1.173	0.247–5.579	0.841	-	-	-
Malperfusion syndrome	1.762	0.765–2.817	0.187	-	-	-
Reaction time ≥5.5 (min)	1.583	0.937–3.092	0.088	-	-	-
Kinetics time ≥1.8 (min)	2.738	1.057–4.758	0.008	2.037	0.932–4.985	0.067
Clot angle <59.4 (deg)	3.300	1.879–7.757	<0.001	4.421	1.922–8.743	<0.001
MA <64.1 (mm)	3.298	1.729–7.285	<0.001	4.552	2.089–8.947	<0.001
Prothrombin time ≥13.0 (sec)	2.474	0.854–7.167	0.095	-	-	-
INR ≥1.1	1.587	0.872–2.888	0.131	-	-	-
Fibrinogen <2.65 (µg/mL)	4.111	2.274–7.433	<0.001	4.473	2.678–9.399	<0.001
D-dimer ≥5.1 (µg/mL)	3.260	1.539–6.908	0.002	2.667	0.925–6.874	0.062
Hemoglobin ≥120.0 (10^9^/L)	0.727	0.357–1.478	0.378	-	-	-
Leukocyte ≥10.0 (10^9^/L)	1.432	0.773–2.652	0.253	-	-	-
Platelet count <201.0 (10^9^/L)	2.197	1.165–4.144	0.015	2.414	0.862–4.565	0.072
Albumin <35.0 (g/L)	1.434	0.771–2.666	0.255	-	-	-
Serum creatinine ≥85.8 (µmol/L)	0.840	0.473–1.492	0.552		-	
Lactic acid ≥1.1 (mmol/L)	1.862	0.941–3.684	0.074			
Alanine aminotransferase ≥43.5 (IU/L)	1.449	0.771–2.722	0.249	-	-	-
Aspartate aminotransferase ≥48.4 (IU/L)	1.774	0.837–3.357	0.146	-	-	-
Operative time ≥320.5 (min)	1.555	0.842–2.870	0.158	-	-	-
CPB time ≥139.5 (min)	3.492	1.921–6.347	<0.001	2.796	1.166–6.705	0.021
Aorta cross-clamp time ≥52.5 (min)	2.745	1.534–4.912	0.001	1.477	0.609–3.579	0.388
SCP time ≥12.8 (min)	1.492	0.841–2.646	0.172	-	-	-
Concomitant procedure	1.541	0.685–2.671	0.255	-	-	-
Intraoperative blood loss ≥290.0 (mL)	1.611	0.692–3.748	0.268	-	-	-
Intraoperative red blood cells transfusion ≥2.5 (U)	2.298	1.284–4.113	0.005	1.651	0.720–3.790	0.237
Intraoperative plasma transfusion ≥200 (mL)	5.090	2.640–9.816	<0.001	4.057	1.700–9.046	0.004

Those factors with *p* < 0.05 in the univariate model were involved in the multivariate model. OR, odds ratio; CI, confidence interval; INR, international normalized ratio; CPB, cardiopulmonary bypass.

### 3.6 Predictive Ability of Risk Factors for Postoperative ARDS

**Fig. 3.**
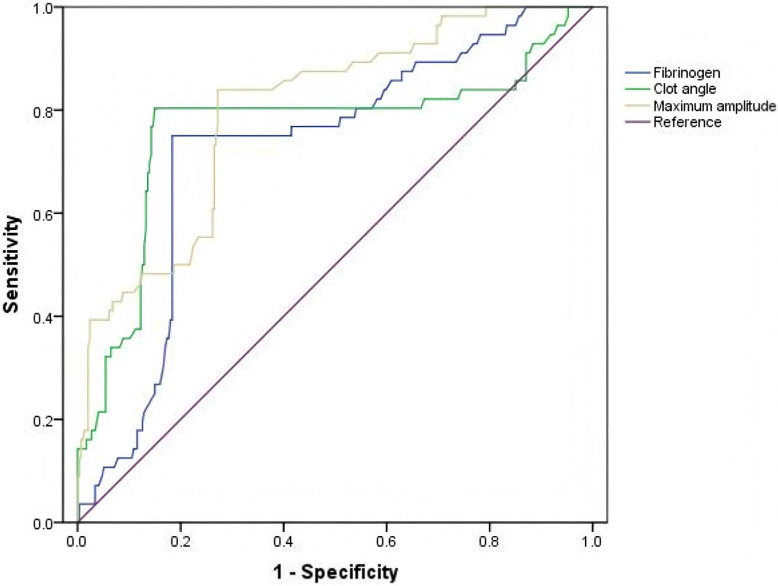
**Value of preoperative fibrinogen and MA levels as predictive risk factors for ARDS in patients with ATAAD based on ROC curve analysis**. ROC, receiver operating characteristic curve.

As shown in Fig. 3, ROC curves were generated to investigate the predictive ability and cut-off value of risk factors for postoperative ARDS in patients with ATAAD. The areas under the curve for preoperative levels of fibrinogen, the clot angle, and MA in the prediction of postoperative ARDS in patients with ATAAD were 0.744 (0.652–0.795, *p* < 0.001; cut-off value, 2.65 µg/mL; sensitivity, 75.0%; specificity, 81.6%; *p* < 0.001), 0.781 (0.675–0.846, *p* < 0.001; cut-off value, 59.4 degrees; sensitivity, 80.4%; specificity, 85.5%; *p* < 0.001), and 0.807 (0.736–0.859, *p* < 0.001; cut-off value, 64.1 mm; sensitivity, 83.9%; specificity, 72.4%; *p* < 0.001), respectively.

## 4. Discussion

The primary finding of this study is that preoperative levels of fibrinogen, the clot angle, and MA are all independent risk factors, with predictive power, for postoperative ARDS in patients with ATAAD. These results further reveal the association between coagulation parameters and the occurrence of ARDS after ATAAD. This study is the first to use TEG to assess the link between coagulation parameters and postoperative ARDS among patients with ATAAD undergoing emergency surgery. Through TEG-based assessments, early detection and the monitoring of individuals at an elevated risk can be accomplished, enabling precise fibrinogen supplementation and improved management of transfusion strategies, thereby providing a scientific basis for the prevention of ARDS and lung-protection strategies following ATAAD.

In this research, the incidence of postoperative ARDS was 16.0% (56/350), and the ARDS group had a longer duration of mechanical ventilation (*p* = 0.009) compared to those in the non-ARDS group. In the ARDS group, 12 patients required reintubation, four developed respiratory failure, and five died. Aortic surgery poses a significant challenge to cardiovascular surgeons owing to its high mortality rate. Severe perioperative bleeding and blood transfusion are among the most frequent and high-risk complications associated with emergency total aortic arch replacement surgery [13, 14]. Large-volume blood transfusion is a known risk factor for postoperative ARDS in patients with ATAAD [15]. Consistent with increasing evidence that perioperative transfusions of large amounts of red blood cells and plasma are independent risk factors for postoperative ARDS [16, 17, 18], in our study, we found that more red blood cells were transfused during surgery in the ARDS group. In addition, studies have shown that the administration of human fibrinogen prior to aortic surgery can reduce the levels of intraoperative blood loss and the extent of transfusion and shorten the operation time effectively. In addition, it can reduce postoperative complications and improve the early prognosis of patients, indicating a relationship between the coagulation system and ARDS [19].

ATAAD itself is associated with a state of coagulation disorder, whereas the findings of Zindovic *et al*. [20] suggested that surgery causes additional damage to the coagulation system in patients with ATAAD. Wang *et al*. [4] further found, through logistic regression models, that the drainage volume 24 h after aortic repair is another independent risk factor for postoperative ARDS. As such, the perioperative coagulation status of patients with ATAAD is a widely accepted clinicopathological indicator of aortic occlusion and CPB-induced coagulopathy during aortic surgery [21]. Prior to emergency aortic surgery, blood contact with the non-endothelial wall of the pseudolumen causes damage to the hemostatic system [21, 22]. Given the activation of the coagulation system before surgery, our research focused on parameters related to the preoperative coagulation system primarily in patients with ATAAD [23]. Routine laboratory tests and TEG allowed for the documentation of early fibrinogen activity, coagulation factor and platelet consumption, and coagulation dysfunction prior to ATAAD. However, to date, no similar research has obtained strong evidence of the relationship between changes in TEG parameters of the coagulation system and postoperative ARDS.

Fibrinogen and platelets are key factors influencing clot formation and strength. Consequently, there has been an increasing emphasis on the role of fibrinogen and platelets in minimizing blood loss and enhancing patient outcomes [24]. Several studies have suggested the use of platelets or fibrinogen to address coagulation dysfunctions [25, 26]. One study also confirmed that fibrinogen and synergistic platelet functions are responsible for the clot strength [27]. Fibrinogen or platelet deficiencies can lead to increased perioperative bleeding, whereas transfusion is associated with postoperative ARDS. In the context of an inflammatory response and coagulopathy, the TEG abnormalities identified in our study likely represent key mechanistic links to the development of ARDS. A low preoperative fibrinogen level reflects the consumption of clotting factors and potentially enhanced fibrinolysis, which may exacerbate endothelial permeability and the release of inflammatory mediators. Similarly, a reduced clot angle indicates impaired fibrin polymerization and cross-linking, leading to an unstable clot that may fail to effectively control local vascular injury and inflammation. A low MA value, reflecting diminished platelet function and its contribution to clot strength, may further exacerbate bleeding tendencies and the need for transfusion, which itself is a potent trigger for inflammatory lung injury. Based on these findings, we propose that changes in TEG parameters associated with postoperative ARDS are predictable and quantifiable in patients with ATAAD.

In addition, when compounded by the systemic insult of CPB and surgery, they may further propel the pathophysiological cascade [17]. This cascade is characterized by amplified inflammation, endothelial damage, and capillary leak, ultimately culminating in ARDS. Owing to the use of extracorporeal circulation in ATAAD surgery, the interaction among nonpulsating blood flow, artificial tubes, the clotting system, and the inflammatory system has been extensively investigated [28, 29, 30]. CPB has also been shown to be associated with an increased incidence of postoperative ARDS [17]. CPB can further lead to the destruction of blood cells and depletion of clotting factors and platelets, with a superimposed effect on the originally collected clotting/fibrinolytic system and platelet activation. CPB may stimulate an inflammatory response, possibly due to blood exposure to abnormal shear forces and contact with artificial duct surfaces [31, 32, 33]. Nteliopoulos *et al*. [34] previously showed that CPB-induced lung injury is caused by multiple factors, including the artificial surface contact of blood components with CPB circuits, local and systemic inflammatory response syndrome, lung ischemia/reperfusion injury, ventilation arrest, and circulating endotoxin production. Further, Liu *et al*. [35] showed that the type of surgery, CPB duration, and blood transfusion are all independent risk factors for postoperative pulmonary complications. It is important to note that the factors that induce ARDS involve different systems that can influence each other. However, conventional laboratory tests can only analyze a single systemic factor, whereas the change in TEG parameters is simple and quantifiable, allowing for an objective and immediate reflection of the overall coagulation state of the body, thus helping to guide clinical practice; optimize blood transfusion; reduce blood transfusion reactions; and shorten hemostasis, CPB, and lung ischemia times to reduce lung damage. As such, the early identification and treatment of clotting systems could be life-saving [36]. In some hospitals, the use of TEG-guided perioperative coagulation management has become common practice to monitor coagulation function and minimize the risk of bleeding [37, 38]. As such, TEG can provide a holistic view of the body’s coagulation system before and after ATAAD and can be used to measure platelet function (MA level) and fibrinogen function (fibrinogen and clot angle levels), which could be advantageous for patients with ATAAD.

### Limitations

This study has several limitations. One of the key limitations is the small sample size from a single center, which may restrict the generalizability of our findings to patients in different settings. Second, because of the hospital conditions, we did not monitor PAWP in patients with ARDS; therefore, there may be a certain diagnostic bias. However, we performed auxiliary tests to compensate for this defect. Third, some potential bias may have been retained following the multivariate analysis. Fourth, this study had a single-center retrospective design. Whereas the sample size was relatively large compared to that of similar studies, it remains limited for analyzing complex surgical outcomes with many potential confounders. Future validation of our findings based on large-scale, multi-center cohorts, with further control for residual confounding, is an excellent direction for future research. Prospective or multi-center collaborative studies are essential to provide more robust evidence of causal associations. Finally, studies with longer follow-ups are required to better understand the relationship between the preoperative coagulation system and development of ARDS after ATAAD.

## 5. Conclusion

Overall, the present study identified preoperative fibrinogen, the clot angle, and MA levels as potential predictors of postoperative ARDS in patients with ATAAD. Further, our results showed that TEG allows for a rapid assessment of the status of the coagulation system in patients with ATAAD before surgery, helping to guide preoperative fibrinogen supplementation and strategic blood transfusion to reduce the occurrence of postoperative ARDS in patients with ATAAD and shorten the postoperative mechanical ventilation time. As such, TEG may be a valuable tool for the real-time monitoring and improvement of patient outcomes after surgery.

## Data Availability

All data generated or analyzed during this study are included in this published article.

## Abbreviations

ATAAD, acute type A aortic dissection; ARDS, acute respiratory distress syndrome; TEG, thromboelastography; MA, maximum amplitude; NYHA, New York Heart Association; LVEF, left ventricular ejection fraction; ECMO, extracorporeal membrane oxygenation; IABP, intra-aortic balloon pump; PaO_2_, arterial partial pressure of oxygen; PAWP, pulmonary artery wedge pressure; OI, oxygen index; CI, confidence interval; OR, odds ratio; SD, standard deviation; ROC, receiver operating characteristic curve; CPB, cardiopulmonary bypass; SCP, selective cerebral perfusion.
